# Supplementary material for: Excess risk of COVID-19 infection and mental distress in healthcare workers during successive pandemic waves: Analysis of matched cohorts of healthcare workers and community referents in Alberta, Canada
Source: Can J Public Health. 2024 Jan 16;115(2):220–9. doi: 10.17269/s41997-023-00848-4 (PMC11006634; doi:10.17269/s41997-023-00848-4)
Supplement: Supplementary file 1 — Supplementary file1 (DOCX 25 KB) [file 41997_2023_848_MOESM1_ESM.docx]

Excess risk of COVID-19 infection and mental distress in healthcare workers during successive pandemic waves: Analysis of matched cohorts of healthcare workers and community referents in Alberta, Canada

Supplementary material

Table 1: Full multiple multilevel piecewise exponential proportional hazards regression

|  | | | | | | | | | | |
| --- | --- | --- | --- | --- | --- | --- | --- | --- | --- | --- |
|  | **From physician record** | | | | | **From PCR test** | | | | |
|  |  |  | **95% CI** | |  |  |  | **95% CI** | |  |
|  | **HR** | **SE** | **Lower** | **Upper** | **p-value** | **HR** | **Std. err.** | **Lower** | **Upper** | **p-value** |
| Baseline hazard for each wave |  |  |  |  |  |  |  |  |  |  |
| Wave 1 | 0.021 | 0.001 | 0.020 | 0.022 | 0.000 | 0.000 | 0.000 | 0.000 | 0.000 | <0.001 |
| Wave 2 | 0.010 | 0.000 | 0.009 | 0.011 | 0.000 | 0.000 | 0.000 | 0.000 | 0.000 | <0.001 |
| Wave 3 | 0.009 | 0.001 | 0.008 | 0.010 | 0.000 | 0.000 | 0.000 | 0.000 | 0.000 | <0.001 |
| Wave 4 | 0.004 | 0.000 | 0.004 | 0.005 | 0.000 | 0.000 | 0.000 | 0.000 | 0.000 | <0.001 |
| Wave 5 | 0.005 | 0.000 | 0.004 | 0.006 | 0.000 | 0.001 | 0.000 | 0.001 | 0.002 | <0.001 |
| HCW relative to controls |  |  |  |  | 0.004* |  |  |  |  | <0.001* |
| Controls | **ref** |  |  |  |  | **ref** |  |  |  |  |
| HCW | 1.309 | 0.158 | 1.033 | 1.658 | 0.026 | 2.452 | 0.255 | 2.001 | 3.005 | <0.001 |
| HCW by Wave |  |  |  |  |  |  |  |  |  |  |
| Controls | **ref** |  |  |  |  | **ref** |  |  |  |  |
| HCW wave 1 | 0.994 | 0.132 | 0.767 | 1.289 | 0.966 | 0.680 | 0.357 | 0.243 | 1.902 | 0.463 |
| HCW wave 2 | 1.002 | 0.138 | 0.764 | 1.313 | 0.991 | 0.208 | 0.038 | 0.146 | 0.297 | <0.001 |
| HCW wave 3 | 0.714 | 0.118 | 0.516 | 0.987 | 0.042 | 0.166 | 0.045 | 0.097 | 0.283 | <0.001 |
| HCW wave 4 | 0.597 | 0.127 | 0.394 | 0.905 | 0.015 | 0.315 | 0.064 | 0.211 | 0.469 | <0.001 |
| HCW wave 5 | **-** |  |  |  |  | **-** |  |  |  |  |
| Vaccine number relative to 0 |  |  |  |  |  |  |  |  |  |  |
| 1 | 1.477 | 0.111 | 1.274 | 1.712 | 0.000 | 0.994 | 0.114 | 0.795 | 1.244 | 0.961 |
| 2 | 1.270 | 0.091 | 1.104 | 1.462 | 0.001 | 0.554 | 0.058 | 0.452 | 0.680 | <0.001 |
| 3 | 1.306 | 0.145 | 1.050 | 1.624 | 0.016 | 0.741 | 0.092 | 0.581 | 0.945 | 0.016 |
| 4+ | 3.802 | 1.734 | 1.555 | 9.294 | 0.003 | 0.316 | 0.270 | 0.059 | 1.687 | 0.178 |
| Number of PCR tests |  |  |  |  |  | 6.446 | 0.189 | 6.085 | 6.828 | <0.001 |
| -Omitted: effectively reduces to the lower order term for HCW relative to controls  *Calculated from a Likelihood ratio test  ref=reference category | | | | | | | | | | |

Example of how to get wave specific HR’s for HCW’s relative to controls from physician record (linear combinations):

$${HR}_{wave1}=\exp\left( \left( \ln\left( 0.021 \right)+\ln\left( 1.309 \right)+ln\left( 0.994 \right) \right)-\ln\left( 0.021 \right) \right)=1.30$$

$${HR}_{wave4}=\exp\left( \left( \ln\left( 0.004 \right)+\ln\left( 1.309 \right)+ln\left( 0.597 \right) \right)-\ln\left( 0.004 \right) \right)=0.78$$
